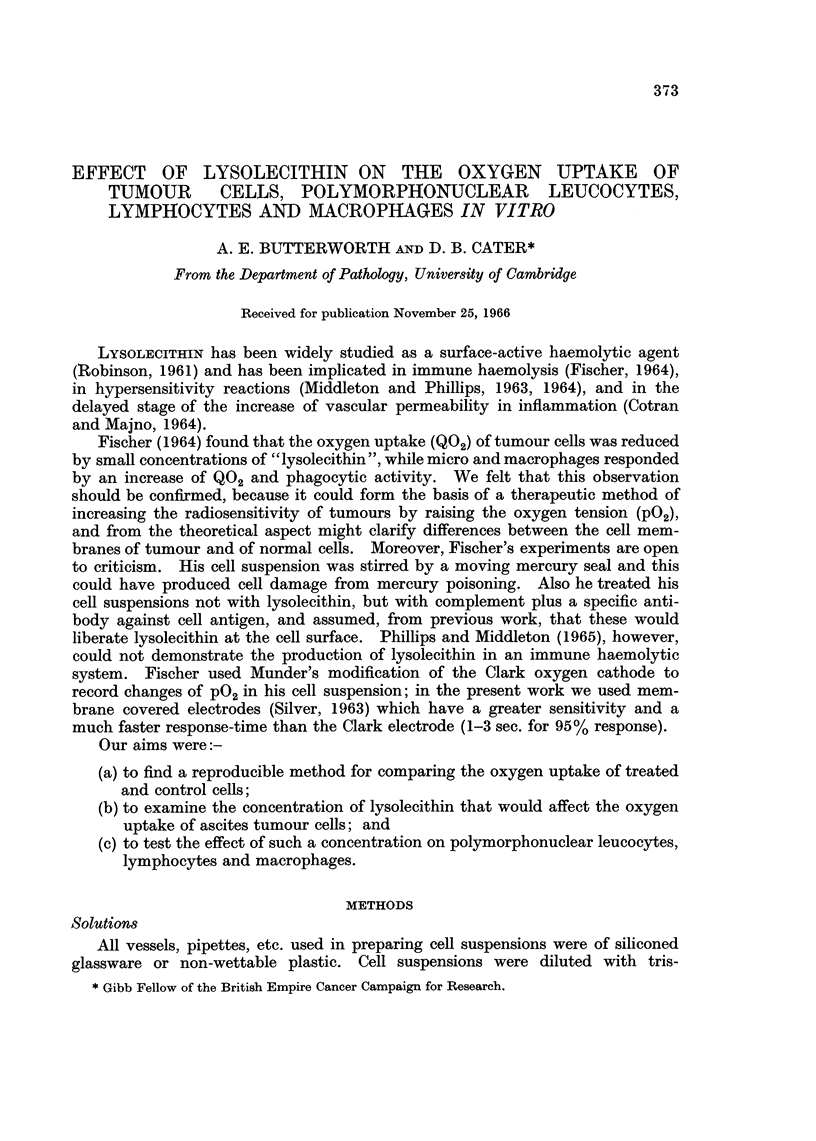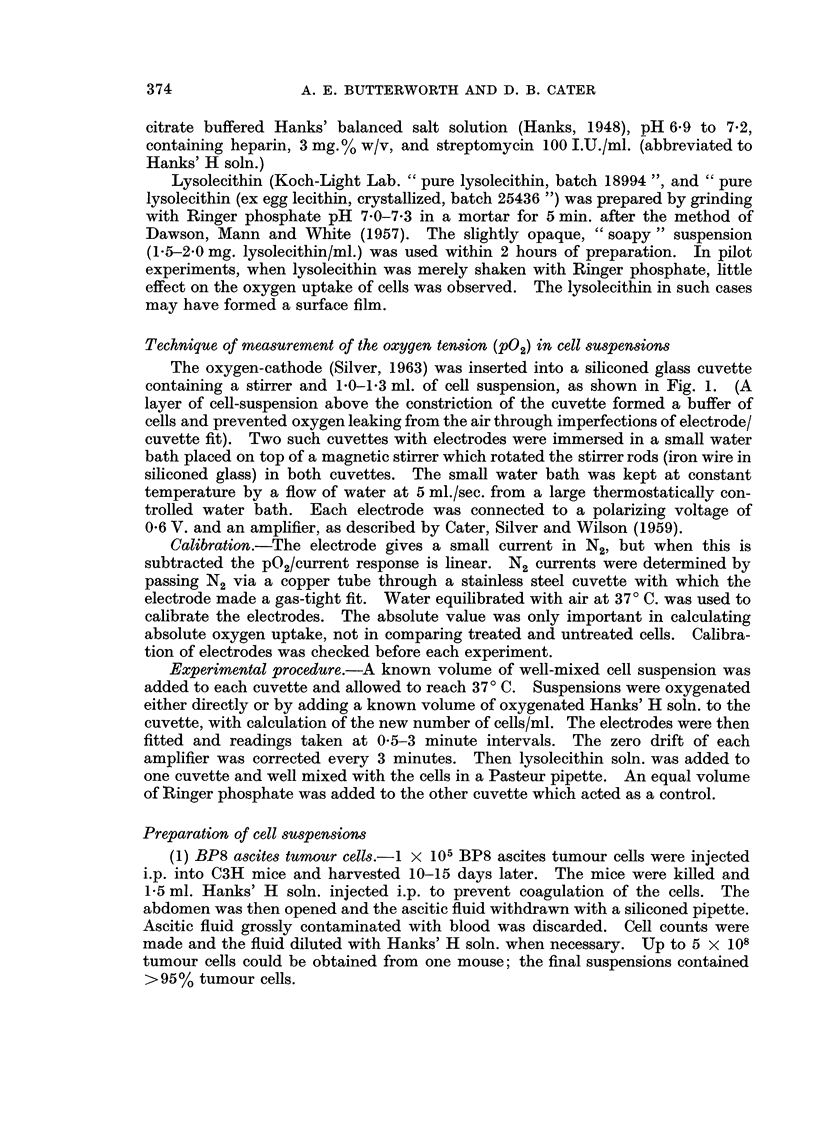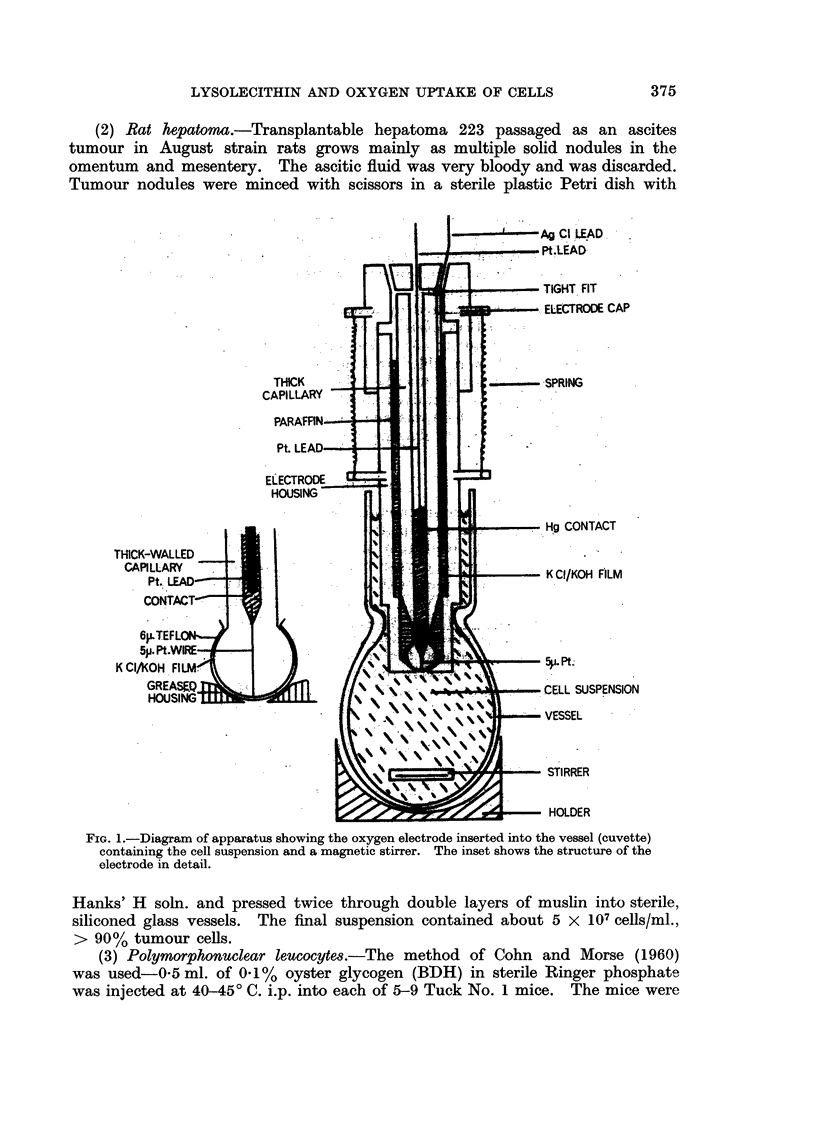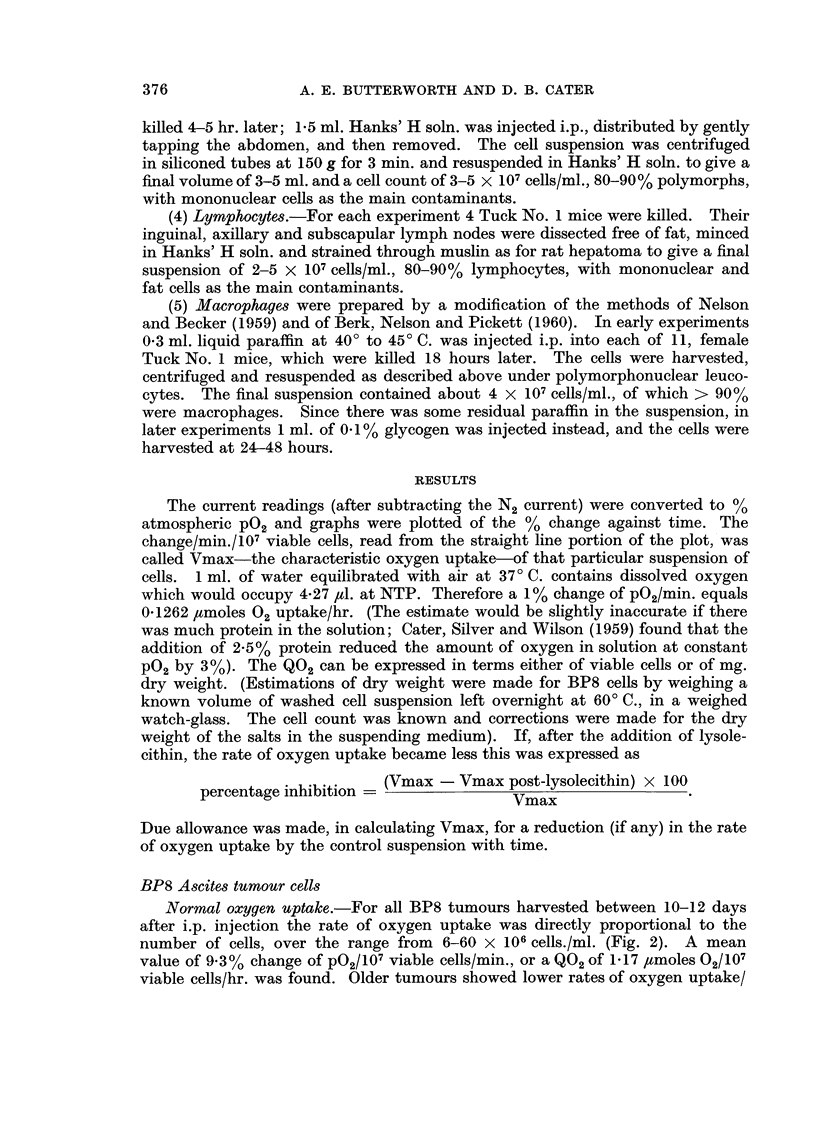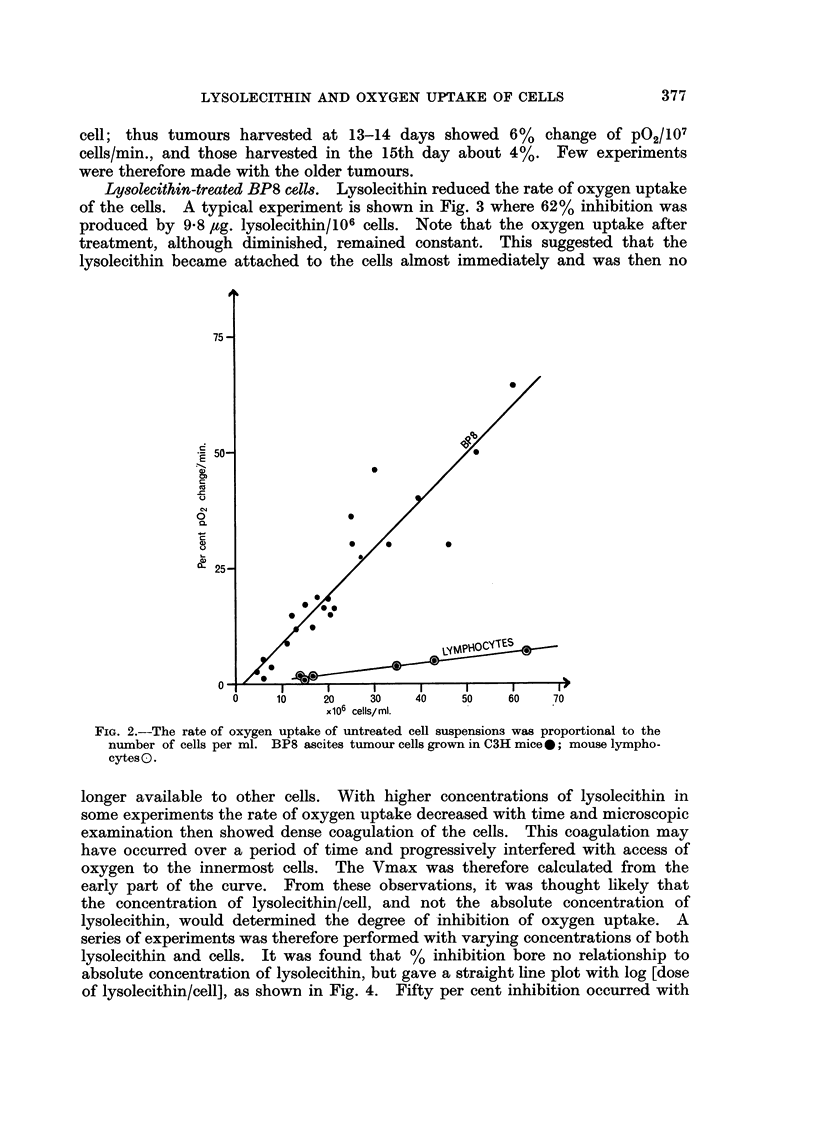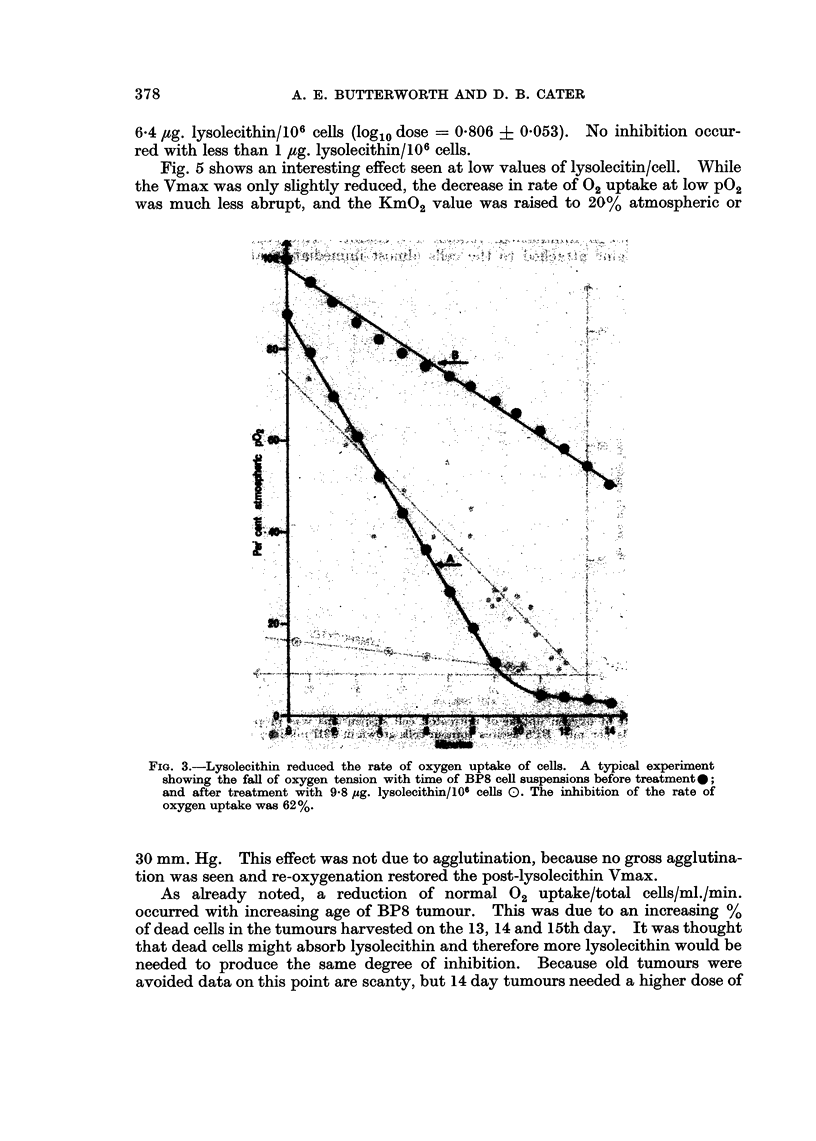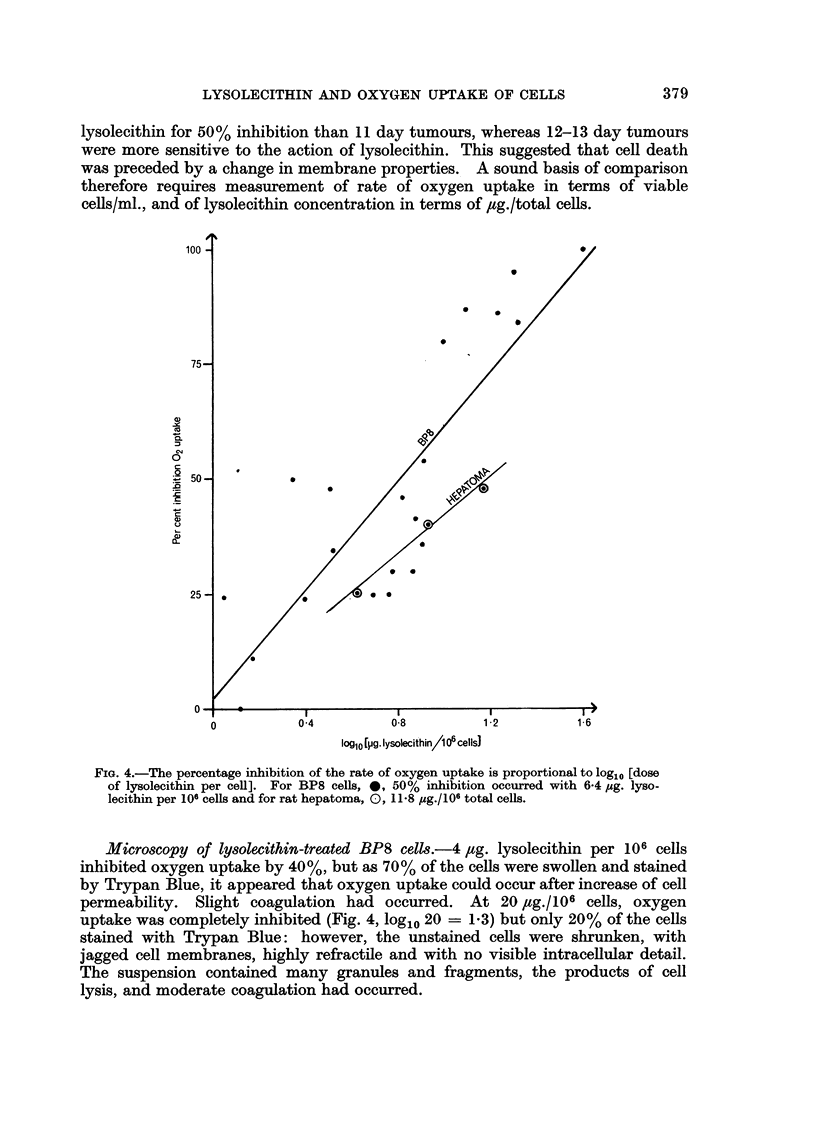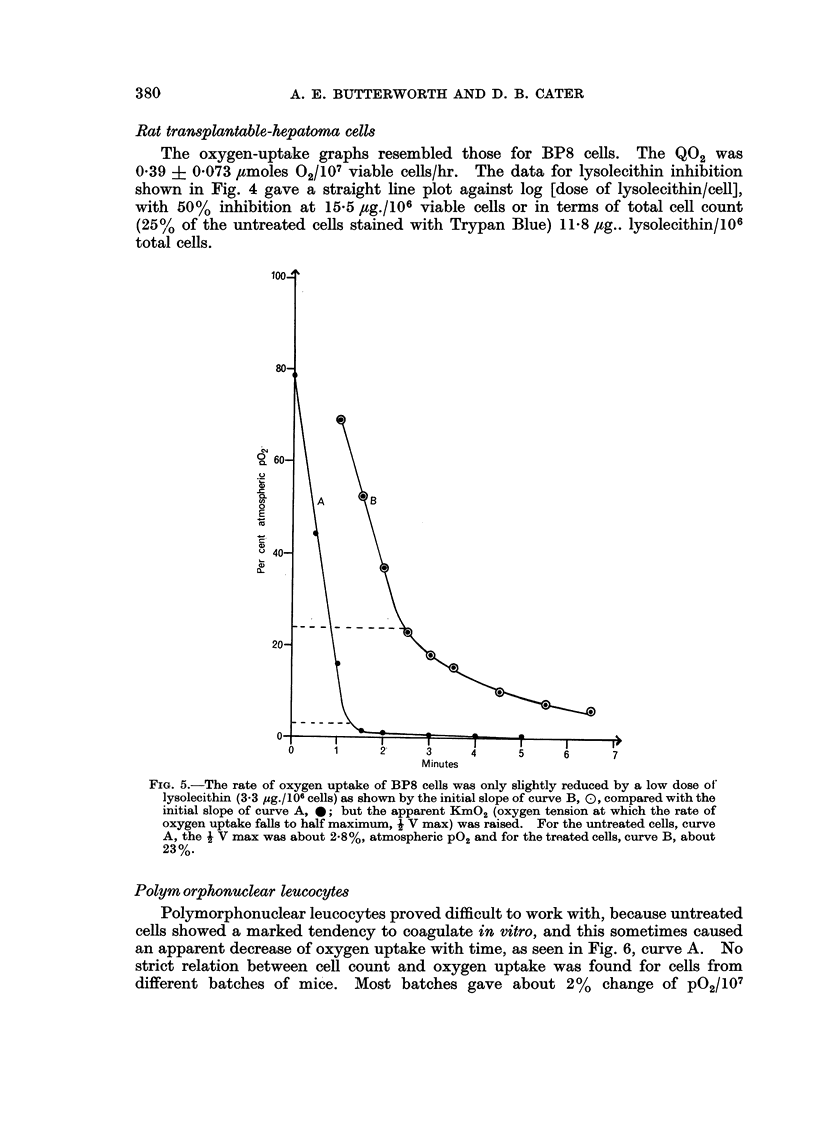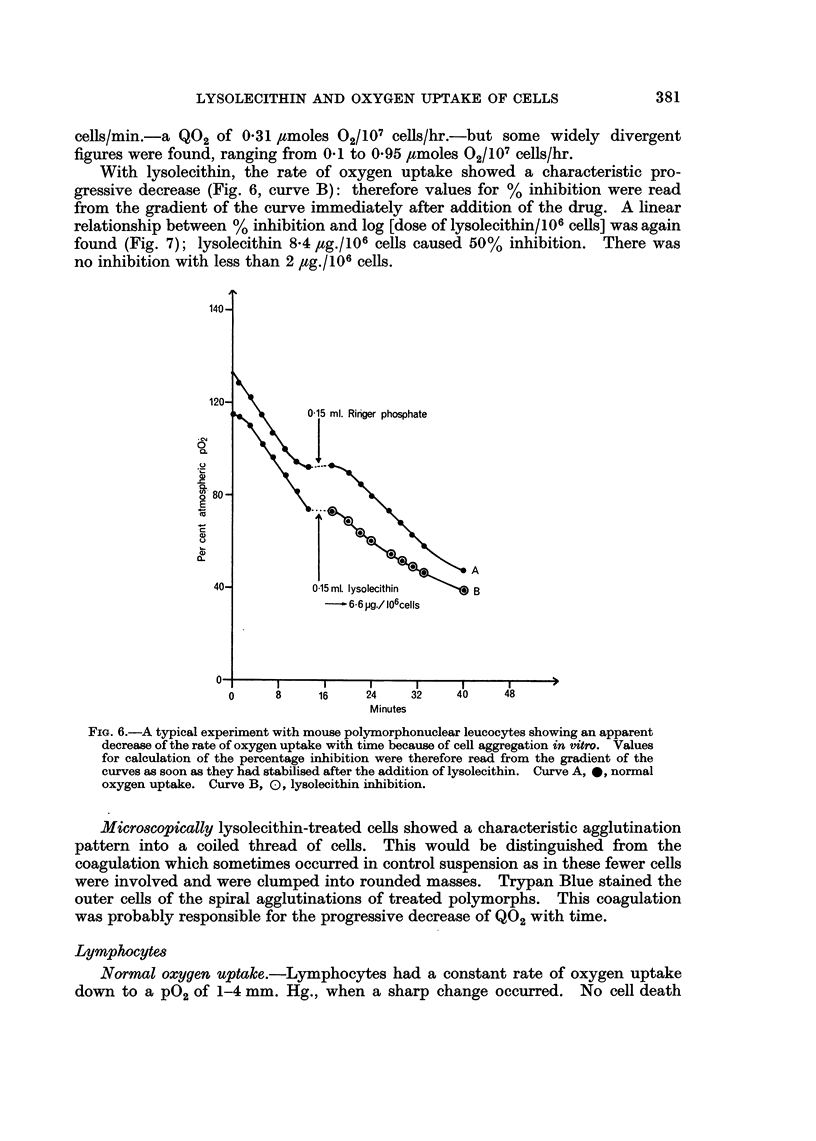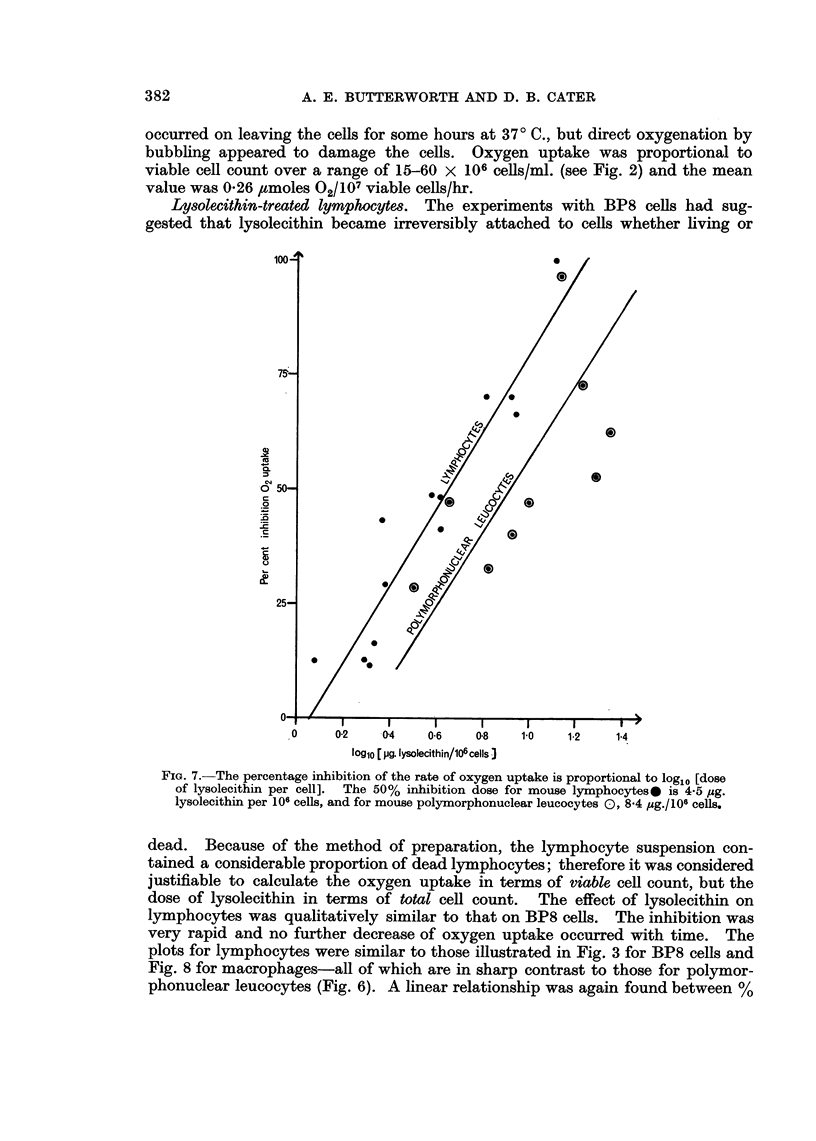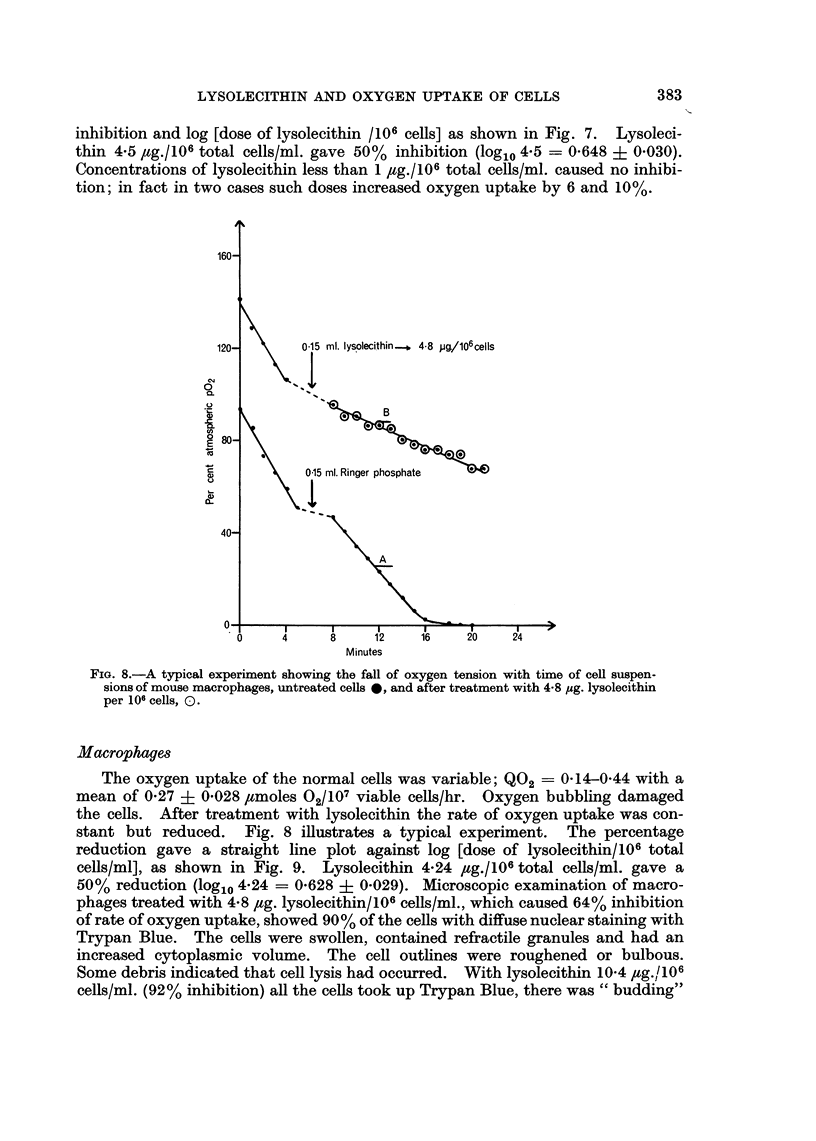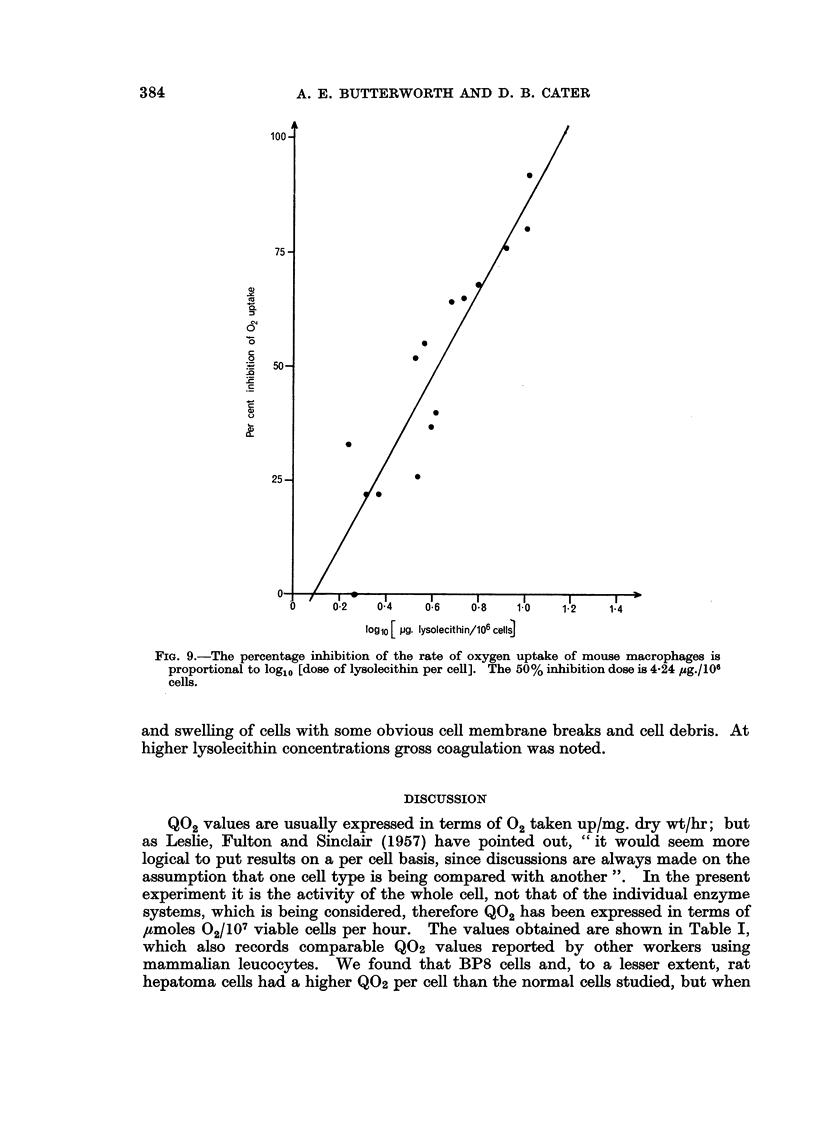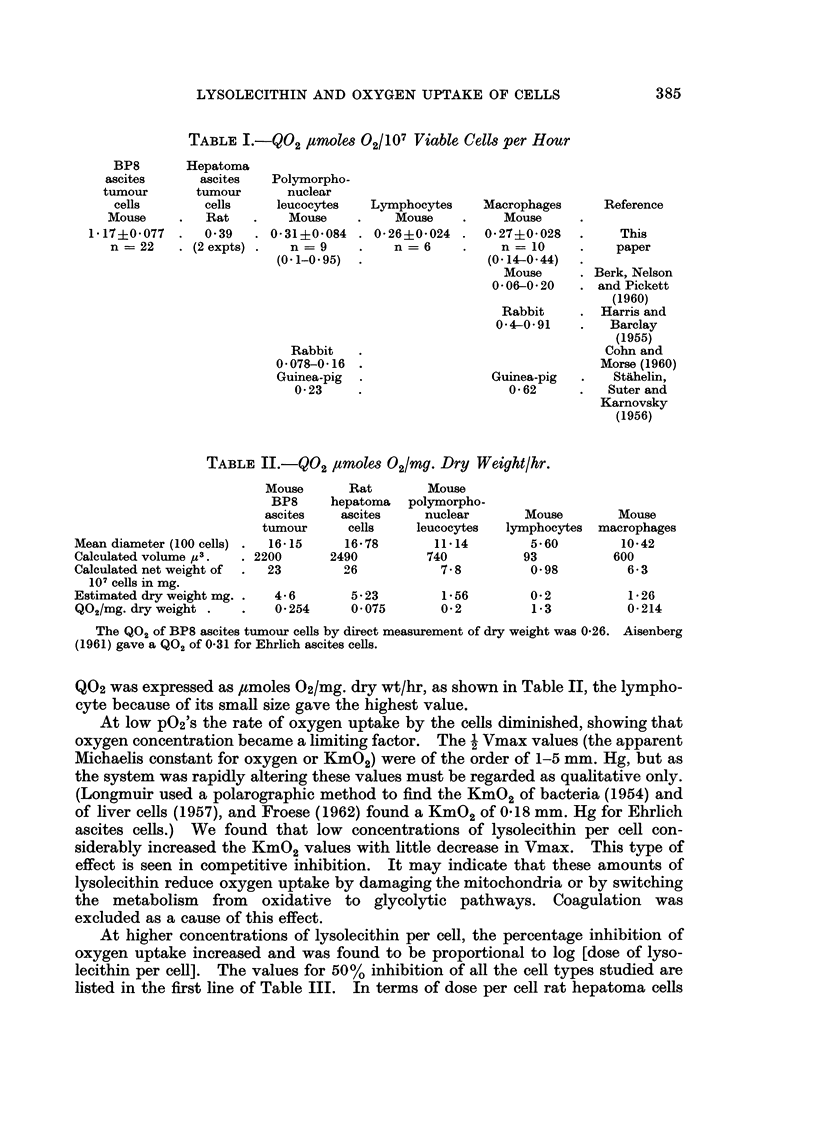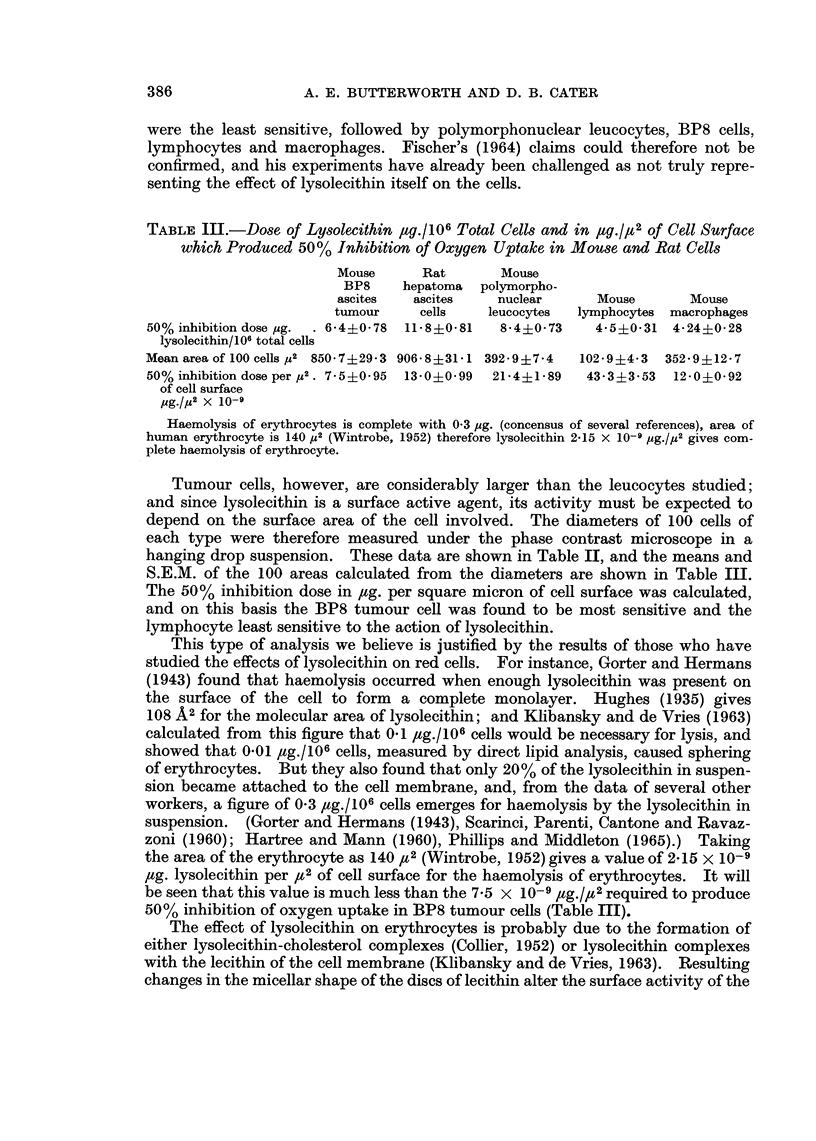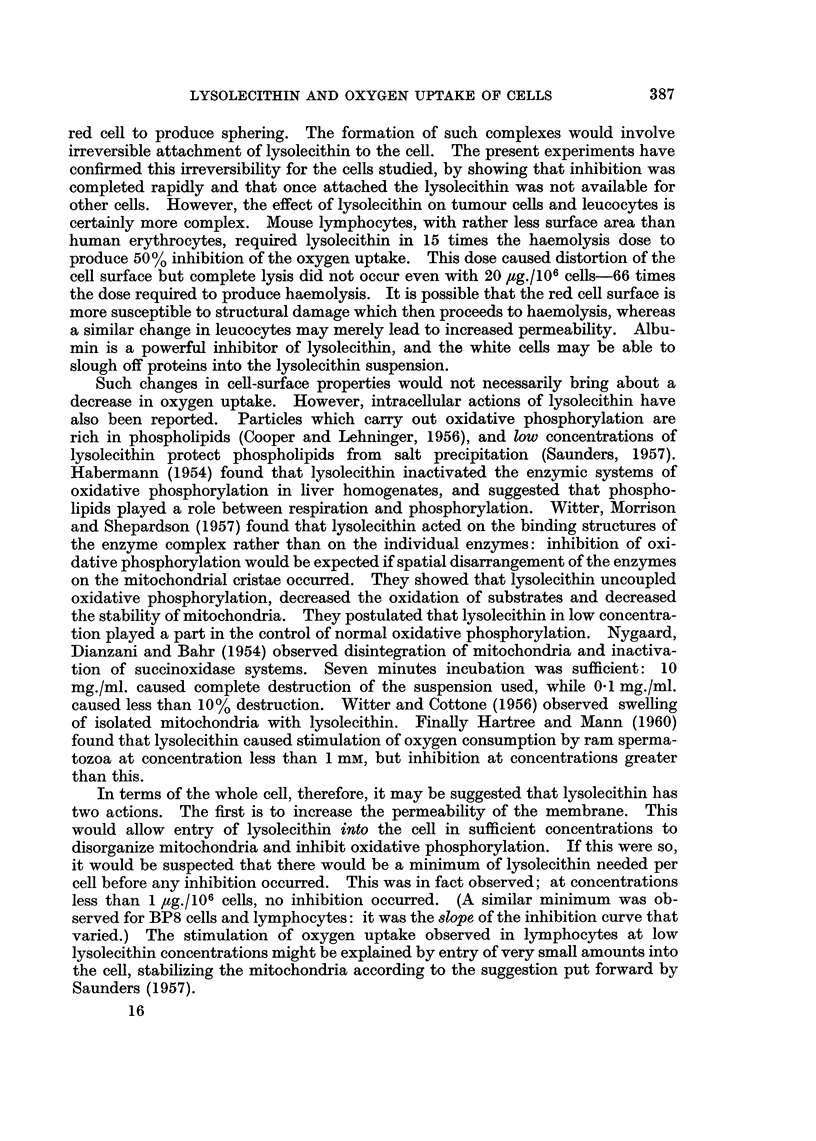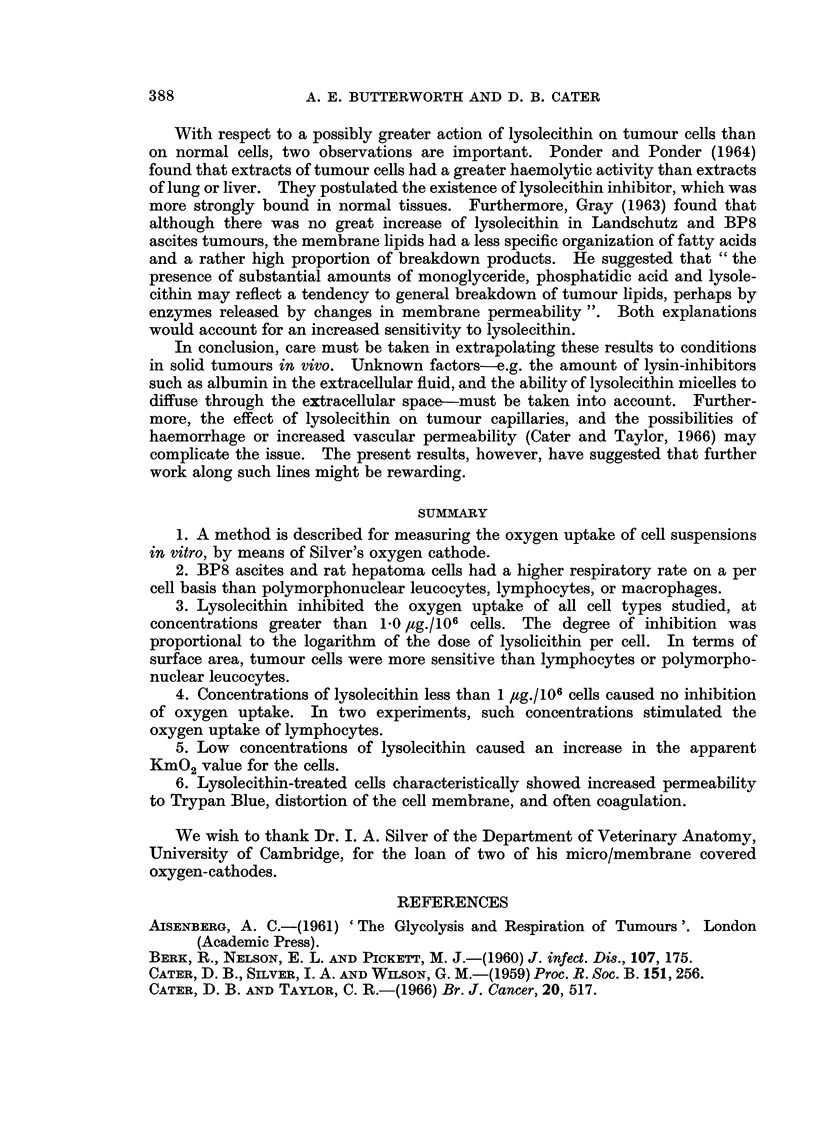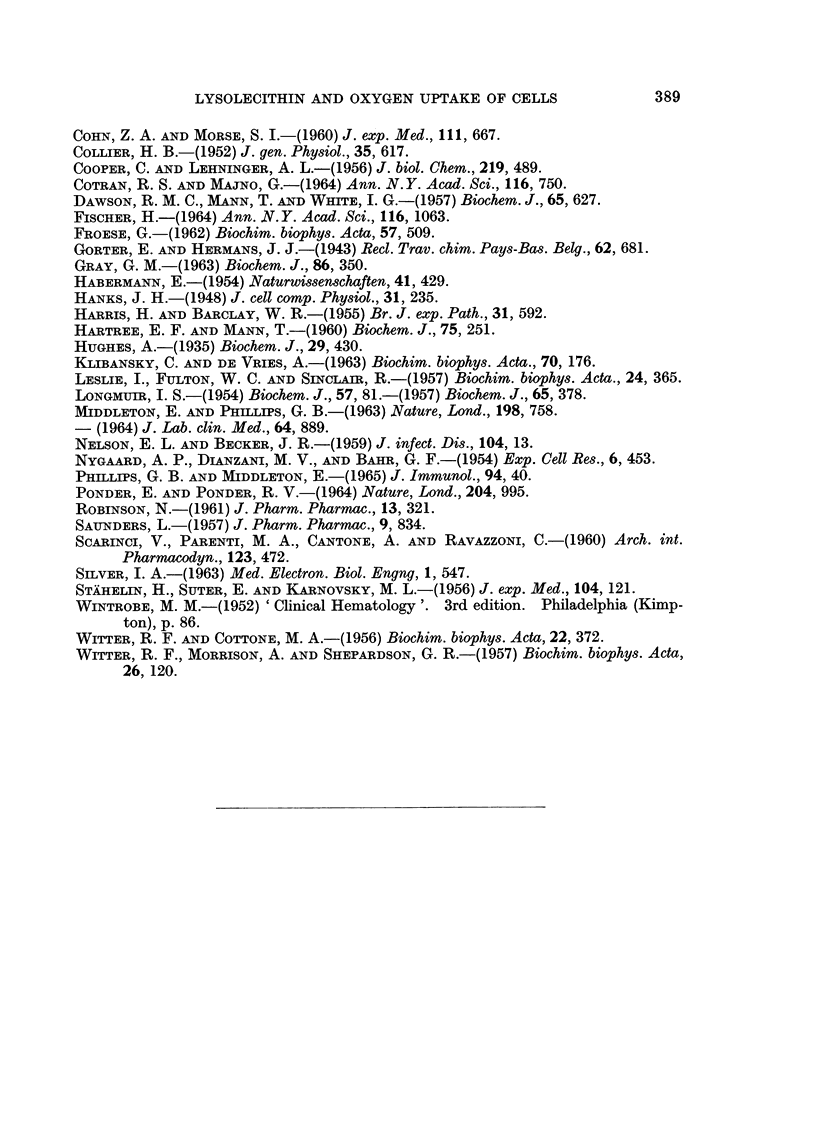# Effect of lysolecithin on the oxygen uptake of tumour cells, polymorphonuclear leucocytes, lymphocytes and macrophages in vitro.

**DOI:** 10.1038/bjc.1967.43

**Published:** 1967-06

**Authors:** A. E. Butterworth, D. B. Cater


### EFFECT OF LYSOLECITHIN ON THE OXYGEN UPTAKE OF TUMOUR CELLS, POLYMORPHONUCLEAR LEUCOCYTES, LYMPHOCYTES AND MACROPHAGES *IN VITRO*

A. E. BUTTERWORTH AND D. B. CATER*

*From the Department of Pathology, University of Cambridge*

Received for publication November 25, 1966

LYSOLECITHIN has been widely studied as a surface-active haemolytic agent (Robinson, 1961) and has been implicated in immune haemolysis (Fischer, 1964), in hypersensitivity reactions (Middleton and Phillips, 1963, 1964), and in the delayed stage of the increase of vascular permeability in inflammation (Cotran and Majno, 1964).

Fischer (1964) found that the oxygen uptake ($QO_2$) of tumour cells was reduced by small concentrations of "lysolecithin", while micro and macrophages responded by an increase of $QO_2$ and phagocytic activity. We felt that this observation should be confirmed, because it could form the basis of a therapeutic method of increasing the radiosensitivity of tumours by raising the oxygen tension ($pO_2$), and from the theoretical aspect might clarify differences between the cell membranes of tumour and of normal cells. Moreover, Fischer's experiments are open to criticism. His cell suspension was stirred by a moving mercury seal and this could have produced cell damage from mercury poisoning. Also he treated his cell suspensions not with lysolecithin, but with complement plus a specific antibody against cell antigen, and assumed, from previous work, that these would liberate lysolecithin at the cell surface. Phillips and Middleton (1965), however, could not demonstrate the production of lysolecithin in an immune haemolytic system. Fischer used Munder's modification of the Clark oxygen cathode to record changes of $pO_2$ in his cell suspension; in the present work we used membrane covered electrodes (Silver, 1963) which have a greater sensitivity and a much faster response-time than the Clark electrode (1–3 sec. for 95% response).

Our aims were:–

(a) to find a reproducible method for comparing the oxygen uptake of treated and control cells;
(b) to examine the concentration of lysolecithin that would affect the oxygen uptake of ascites tumour cells; and
(c) to test the effect of such a concentration on polymorphonuclear leucocytes, lymphocytes and macrophages.

### METHODS

### *Solutions*

All vessels, pipettes, etc. used in preparing cell suspensions were of siliconed glassware or non-wettable plastic. Cell suspensions were diluted with tris-

* Gibb Fellow of the British Empire Cancer Campaign for Research.

citrate buffered Hanks' balanced salt solution (Hanks, 1948), pH 6·9 to 7·2, containing heparin, 3 mg.% w/v, and streptomycin 100 I.U./ml. (abbreviated to Hanks' H soln.)

Lysolecithin (Koch-Light Lab. " pure lysolecithin, batch 18994 ", and " pure lysolecithin (ex egg lecithin, crystallized, batch 25436 ") was prepared by grinding with Ringer phosphate pH 7·0–7·3 in a mortar for 5 min. after the method of Dawson, Mann and White (1957). The slightly opaque, " soapy " suspension (1·5–2·0 mg. lysolecithin/ml.) was used within 2 hours of preparation. In pilot experiments, when lysolecithin was merely shaken with Ringer phosphate, little effect on the oxygen uptake of cells was observed. The lysolecithin in such cases may have formed a surface film.

*Technique of measurement of the oxygen tension ($pO_2$) in cell suspensions*

The oxygen-cathode (Silver, 1963) was inserted into a siliconed glass cuvette containing a stirrer and 1·0–1·3 ml. of cell suspension, as shown in Fig. 1. (A layer of cell-suspension above the constriction of the cuvette formed a buffer of cells and prevented oxygen leaking from the air through imperfections of electrode/cuvette fit). Two such cuvettes with electrodes were immersed in a small water bath placed on top of a magnetic stirrer which rotated the stirrer rods (iron wire in siliconed glass) in both cuvettes. The small water bath was kept at constant temperature by a flow of water at 5 ml./sec. from a large thermostatically controlled water bath. Each electrode was connected to a polarizing voltage of 0·6 V. and an amplifier, as described by Cater, Silver and Wilson (1959).

*Calibration.*—The electrode gives a small current in $N_2$, but when this is subtracted the $pO_2$/current response is linear. $N_2$ currents were determined by passing $N_2$ via a copper tube through a stainless steel cuvette with which the electrode made a gas-tight fit. Water equilibrated with air at 37° C. was used to calibrate the electrodes. The absolute value was only important in calculating absolute oxygen uptake, not in comparing treated and untreated cells. Calibration of electrodes was checked before each experiment.

*Experimental procedure.*—A known volume of well-mixed cell suspension was added to each cuvette and allowed to reach 37° C. Suspensions were oxygenated either directly or by adding a known volume of oxygenated Hanks' H soln. to the cuvette, with calculation of the new number of cells/ml. The electrodes were then fitted and readings taken at 0·5–3 minute intervals. The zero drift of each amplifier was corrected every 3 minutes. Then lysolecithin soln. was added to one cuvette and well mixed with the cells in a Pasteur pipette. An equal volume of Ringer phosphate was added to the other cuvette which acted as a control.

*Preparation of cell suspensions*

(1) *BP8 ascites tumour cells.*—$1 \times 10^5$ BP8 ascites tumour cells were injected i.p. into C3H mice and harvested 10–15 days later. The mice were killed and 1·5 ml. Hanks' H soln. injected i.p. to prevent coagulation of the cells. The abdomen was then opened and the ascitic fluid withdrawn with a siliconed pipette. Ascitic fluid grossly contaminated with blood was discarded. Cell counts were made and the fluid diluted with Hanks' H soln. when necessary. Up to $5 \times 10^8$ tumour cells could be obtained from one mouse; the final suspensions contained $>95\%$ tumour cells.

(2) *Rat hepatoma.*—Transplantable hepatoma 223 passaged as an ascites tumour in August strain rats grows mainly as multiple solid nodules in the omentum and mesentery. The ascitic fluid was very bloody and was discarded. Tumour nodules were minced with scissors in a sterile plastic Petri dish with

**Fig. 1.—Diagram of apparatus showing the oxygen electrode inserted into the vessel (cuvette) containing the cell suspension and a magnetic stirrer. The inset shows the structure of the electrode in detail.**

Hanks' H soln. and pressed twice through double layers of muslin into sterile, siliconed glass vessels. The final suspension contained about $5 \times 10^7$ cells/ml., $> 90\%$ tumour cells.

(3) *Polymorphonuclear leucocytes.*—The method of Cohn and Morse (1960) was used—0·5 ml. of 0·1% oyster glycogen (BDH) in sterile Ringer phosphate was injected at 40–45° C. i.p. into each of 5–9 Tuck No. 1 mice. The mice were killed 4–5 hr. later; 1·5 ml. Hanks' H soln. was injected i.p., distributed by gently tapping the abdomen, and then removed. The cell suspension was centrifuged in siliconed tubes at 150 g for 3 min. and resuspended in Hanks' H soln. to give a final volume of 3–5 ml. and a cell count of 3–5 $\times 10^7$ cells/ml., 80–90% polymorphs, with mononuclear cells as the main contaminants.

(4) *Lymphocytes.*—For each experiment 4 Tuck No. 1 mice were killed. Their inguinal, axillary and subscapular lymph nodes were dissected free of fat, minced in Hanks' H soln. and strained through muslin as for rat hepatoma to give a final suspension of 2–5 $\times 10^7$ cells/ml., 80–90% lymphocytes, with mononuclear and fat cells as the main contaminants.

(5) *Macrophages* were prepared by a modification of the methods of Nelson and Becker (1959) and of Berk, Nelson and Pickett (1960). In early experiments 0·3 ml. liquid paraffin at 40° to 45° C. was injected i.p. into each of 11, female Tuck No. 1 mice, which were killed 18 hours later. The cells were harvested, centrifuged and resuspended as described above under polymorphonuclear leucocytes. The final suspension contained about 4 $\times 10^7$ cells/ml., of which > 90% were macrophages. Since there was some residual paraffin in the suspension, in later experiments 1 ml. of 0·1% glycogen was injected instead, and the cells were harvested at 24–48 hours.

### RESULTS

The current readings (after subtracting the $N_2$ current) were converted to % atmospheric $pO_2$ and graphs were plotted of the % change against time. The change/min./$10^7$ viable cells, read from the straight line portion of the plot, was called Vmax—the characteristic oxygen uptake—of that particular suspension of cells. 1 ml. of water equilibrated with air at 37° C. contains dissolved oxygen which would occupy 4·27 $\mu$l. at NTP. Therefore a 1% change of $pO_2$/min. equals 0·1262 $\mu$moles $O_2$ uptake/hr. (The estimate would be slightly inaccurate if there was much protein in the solution; Cater, Silver and Wilson (1959) found that the addition of 2·5% protein reduced the amount of oxygen in solution at constant $pO_2$ by 3%). The $QO_2$ can be expressed in terms either of viable cells or of mg. dry weight. (Estimations of dry weight were made for BP8 cells by weighing a known volume of washed cell suspension left overnight at 60° C., in a weighed watch-glass. The cell count was known and corrections were made for the dry weight of the salts in the suspending medium). If, after the addition of lysolecithin, the rate of oxygen uptake became less this was expressed as

$$\text{percentage inhibition} = \frac{(\text{Vmax} - \text{Vmax post-lysolecithin}) \times 100}{\text{Vmax}}.$$

Due allowance was made, in calculating Vmax, for a reduction (if any) in the rate of oxygen uptake by the control suspension with time.

### *BP8 Ascites tumour cells*

*Normal oxygen uptake.*—For all BP8 tumours harvested between 10–12 days after i.p. injection the rate of oxygen uptake was directly proportional to the number of cells, over the range from 6–60 $\times 10^6$ cells./ml. (Fig. 2). A mean value of 9·3% change of $pO_2/10^7$ viable cells/min., or a $QO_2$ of 1·17 $\mu$moles $O_2/10^7$ viable cells/hr. was found. Older tumours showed lower rates of oxygen uptake/

cell; thus tumours harvested at 13–14 days showed 6% change of $pO_2/10^7$ cells/min., and those harvested in the 15th day about 4%. Few experiments were therefore made with the older tumours.

*Lysolecithin-treated BP8 cells.* Lysolecithin reduced the rate of oxygen uptake of the cells. A typical experiment is shown in Fig. 3 where 62% inhibition was produced by 9·8 μg. lysolecithin/$10^6$ cells. Note that the oxygen uptake after treatment, although diminished, remained constant. This suggested that the lysolecithin became attached to the cells almost immediately and was then no

FIG. 2.—The rate of oxygen uptake of untreated cell suspensions was proportional to the number of cells per ml. BP8 ascites tumour cells grown in C3H mice ●; mouse lymphocytes ○.

longer available to other cells. With higher concentrations of lysolecithin in some experiments the rate of oxygen uptake decreased with time and microscopic examination then showed dense coagulation of the cells. This coagulation may have occurred over a period of time and progressively interfered with access of oxygen to the innermost cells. The Vmax was therefore calculated from the early part of the curve. From these observations, it was thought likely that the concentration of lysolecithin/cell, and not the absolute concentration of lysolecithin, would determined the degree of inhibition of oxygen uptake. A series of experiments was therefore performed with varying concentrations of both lysolecithin and cells. It was found that % inhibition bore no relationship to absolute concentration of lysolecithin, but gave a straight line plot with log [dose of lysolecithin/cell], as shown in Fig. 4. Fifty per cent inhibition occurred with

6·4 $\mu g$. lysolecithin/$10^6$ cells ($\log_{10}$ dose $= 0{\cdot}806 \pm 0{\cdot}053$). No inhibition occurred with less than 1 $\mu g$. lysolecithin/$10^6$ cells.

Fig. 5 shows an interesting effect seen at low values of lysolecitin/cell. While the Vmax was only slightly reduced, the decrease in rate of $O_2$ uptake at low $pO_2$ was much less abrupt, and the $KmO_2$ value was raised to 20% atmospheric or

FIG. 3.—Lysolecithin reduced the rate of oxygen uptake of cells. A typical experiment showing the fall of oxygen tension with time of BP8 cell suspensions before treatment ●; and after treatment with 9·8 $\mu g$. lysolecithin/$10^6$ cells ⊙. The inhibition of the rate of oxygen uptake was 62%.

30 mm. Hg. This effect was not due to agglutination, because no gross agglutination was seen and re-oxygenation restored the post-lysolecithin Vmax.

As already noted, a reduction of normal $O_2$ uptake/total cells/ml./min. occurred with increasing age of BP8 tumour. This was due to an increasing % of dead cells in the tumours harvested on the 13, 14 and 15th day. It was thought that dead cells might absorb lysolecithin and therefore more lysolecithin would be needed to produce the same degree of inhibition. Because old tumours were avoided data on this point are scanty, but 14 day tumours needed a higher dose of lysolecithin for 50% inhibition than 11 day tumours, whereas 12–13 day tumours were more sensitive to the action of lysolecithin. This suggested that cell death was preceded by a change in membrane properties. A sound basis of comparison therefore requires measurement of rate of oxygen uptake in terms of viable cells/ml., and of lysolecithin concentration in terms of $\mu$g./total cells.

FIG. 4.—The percentage inhibition of the rate of oxygen uptake is proportional to $\log_{10}$ [dose of lysolecithin per cell]. For BP8 cells, ●, 50% inhibition occurred with 6·4 $\mu$g. lysolecithin per $10^6$ cells and for rat hepatoma, ⊙, 11·8 $\mu$g./$10^6$ total cells.

*Microscopy of lysolecithin-treated BP*8 *cells*.—4 $\mu$g. lysolecithin per $10^6$ cells inhibited oxygen uptake by 40%, but as 70% of the cells were swollen and stained by Trypan Blue, it appeared that oxygen uptake could occur after increase of cell permeability. Slight coagulation had occurred. At 20 $\mu$g./$10^6$ cells, oxygen uptake was completely inhibited (Fig. 4, $\log_{10} 20 = 1 \cdot 3$) but only 20% of the cells stained with Trypan Blue: however, the unstained cells were shrunken, with jagged cell membranes, highly refractile and with no visible intracellular detail. The suspension contained many granules and fragments, the products of cell lysis, and moderate coagulation had occurred.

*Rat transplantable-hepatoma cells*

The oxygen-uptake graphs resembled those for BP8 cells. The $QO_2$ was 0·39 ± 0·073 μmoles $O_2/10^7$ viable cells/hr. The data for lysolecithin inhibition shown in Fig. 4 gave a straight line plot against log [dose of lysolecithin/cell], with 50% inhibition at 15·5 μg./$10^6$ viable cells or in terms of total cell count (25% of the untreated cells stained with Trypan Blue) 11·8 μg.. lysolecithin/$10^6$ total cells.

FIG. 5.—The rate of oxygen uptake of BP8 cells was only slightly reduced by a low dose of lysolecithin (3·3 μg./$10^6$ cells) as shown by the initial slope of curve B, ⊙, compared with the initial slope of curve A, ●; but the apparent $KmO_2$ (oxygen tension at which the rate of oxygen uptake falls to half maximum, ½ V max) was raised. For the untreated cells, curve A, the ½ V max was about 2·8%, atmospheric $pO_2$ and for the treated cells, curve B, about 23%.

*Polym orphonuclear leucocytes*

Polymorphonuclear leucocytes proved difficult to work with, because untreated cells showed a marked tendency to coagulate *in vitro*, and this sometimes caused an apparent decrease of oxygen uptake with time, as seen in Fig. 6, curve A. No strict relation between cell count and oxygen uptake was found for cells from different batches of mice. Most batches gave about 2% change of $pO_2/10^7$

cells/min.—a $QO_2$ of 0·31 $\mu$moles $O_2/10^7$ cells/hr.—but some widely divergent figures were found, ranging from 0·1 to 0·95 $\mu$moles $O_2/10^7$ cells/hr.

With lysolecithin, the rate of oxygen uptake showed a characteristic progressive decrease (Fig. 6, curve B): therefore values for % inhibition were read from the gradient of the curve immediately after addition of the drug. A linear relationship between % inhibition and log [dose of lysolecithin/$10^6$ cells] was again found (Fig. 7); lysolecithin 8·4 $\mu$g./$10^6$ cells caused 50% inhibition. There was no inhibition with less than 2 $\mu$g./$10^6$ cells.

FIG. 6.—A typical experiment with mouse polymorphonuclear leucocytes showing an apparent decrease of the rate of oxygen uptake with time because of cell aggregation *in vitro*. Values for calculation of the percentage inhibition were therefore read from the gradient of the curves as soon as they had stabilised after the addition of lysolecithin. Curve A, ●, normal oxygen uptake. Curve B, ⊙, lysolecithin inhibition.

*Microscopically* lysolecithin-treated cells showed a characteristic agglutination pattern into a coiled thread of cells. This would be distinguished from the coagulation which sometimes occurred in control suspension as in these fewer cells were involved and were clumped into rounded masses. Trypan Blue stained the outer cells of the spiral agglutinations of treated polymorphs. This coagulation was probably responsible for the progressive decrease of $QO_2$ with time.

*Lymphocytes*

*Normal oxygen uptake.*—Lymphocytes had a constant rate of oxygen uptake down to a $pO_2$ of 1–4 mm. Hg., when a sharp change occurred. No cell death occurred on leaving the cells for some hours at 37° C., but direct oxygenation by bubbling appeared to damage the cells. Oxygen uptake was proportional to viable cell count over a range of 15–60 × $10^6$ cells/ml. (see Fig. 2) and the mean value was 0·26 $\mu$moles $O_2/10^7$ viable cells/hr.

*Lysolecithin-treated lymphocytes.* The experiments with BP8 cells had suggested that lysolecithin became irreversibly attached to cells whether living or

FIG. 7.—The percentage inhibition of the rate of oxygen uptake is proportional to $\log_{10}$ [dose of lysolecithin per cell]. The 50% inhibition dose for mouse lymphocytes ● is 4·5 $\mu$g. lysolecithin per $10^6$ cells, and for mouse polymorphonuclear leucocytes ⊙, 8·4 $\mu$g./$10^6$ cells.

dead. Because of the method of preparation, the lymphocyte suspension contained a considerable proportion of dead lymphocytes; therefore it was considered justifiable to calculate the oxygen uptake in terms of *viable* cell count, but the dose of lysolecithin in terms of *total* cell count. The effect of lysolecithin on lymphocytes was qualitatively similar to that on BP8 cells. The inhibition was very rapid and no further decrease of oxygen uptake occurred with time. The plots for lymphocytes were similar to those illustrated in Fig. 3 for BP8 cells and Fig. 8 for macrophages—all of which are in sharp contrast to those for polymorphonuclear leucocytes (Fig. 6). A linear relationship was again found between %

inhibition and log [dose of lysolecithin /$10^6$ cells] as shown in Fig. 7. Lysolecithin 4·5 μg./$10^6$ total cells/ml. gave 50% inhibition ($\log_{10}$ 4·5 = 0·648 ± 0·030). Concentrations of lysolecithin less than 1 μg./$10^6$ total cells/ml. caused no inhibition; in fact in two cases such doses increased oxygen uptake by 6 and 10%.

FIG. 8.—A typical experiment showing the fall of oxygen tension with time of cell suspensions of mouse macrophages, untreated cells ●, and after treatment with 4·8 μg. lysolecithin per $10^6$ cells, ⊙.

*Macrophages*

The oxygen uptake of the normal cells was variable; $QO_2$ = 0·14–0·44 with a mean of 0·27 ± 0·028 μmoles $O_2/10^7$ viable cells/hr. Oxygen bubbling damaged the cells. After treatment with lysolecithin the rate of oxygen uptake was constant but reduced. Fig. 8 illustrates a typical experiment. The percentage reduction gave a straight line plot against log [dose of lysolecithin/$10^6$ total cells/ml], as shown in Fig. 9. Lysolecithin 4·24 μg./$10^6$ total cells/ml. gave a 50% reduction ($\log_{10}$ 4·24 = 0·628 ± 0·029). Microscopic examination of macrophages treated with 4·8 μg. lysolecithin/$10^6$ cells/ml., which caused 64% inhibition of rate of oxygen uptake, showed 90% of the cells with diffuse nuclear staining with Trypan Blue. The cells were swollen, contained refractile granules and had an increased cytoplasmic volume. The cell outlines were roughened or bulbous. Some debris indicated that cell lysis had occurred. With lysolecithin 10·4 μg./$10^6$ cells/ml. (92% inhibition) all the cells took up Trypan Blue, there was " budding"

FIG. 9.—The percentage inhibition of the rate of oxygen uptake of mouse macrophages is proportional to $\log_{10}$ [dose of lysolecithin per cell]. The 50% inhibition dose is 4·24 $\mu$g./$10^6$ cells.

and swelling of cells with some obvious cell membrane breaks and cell debris. At higher lysolecithin concentrations gross coagulation was noted.

### DISCUSSION

$QO_2$ values are usually expressed in terms of $O_2$ taken up/mg. dry wt/hr; but as Leslie, Fulton and Sinclair (1957) have pointed out, " it would seem more logical to put results on a per cell basis, since discussions are always made on the assumption that one cell type is being compared with another ". In the present experiment it is the activity of the whole cell, not that of the individual enzyme systems, which is being considered, therefore $QO_2$ has been expressed in terms of $\mu$moles $O_2/10^7$ viable cells per hour. The values obtained are shown in Table I, which also records comparable $QO_2$ values reported by other workers using mammalian leucocytes. We found that BP8 cells and, to a lesser extent, rat hepatoma cells had a higher $QO_2$ per cell than the normal cells studied, but when

TABLE I.—*$QO_2$ µmoles $O_2/10^7$ Viable Cells per Hour*

| BP8 ascites tumour cells Mouse | Hepatoma ascites tumour cells Rat | Polymorphonuclear leucocytes Mouse | Lymphocytes Mouse | Macrophages Mouse | Reference |
|---|---|---|---|---|---|
| $1 \cdot 17 \pm 0 \cdot 077$ n = 22 | 0·39 (2 expts) | $0 \cdot 31 \pm 0 \cdot 084$ n = 9 (0·1–0·95) | $0 \cdot 26 \pm 0 \cdot 024$ n = 6 | $0 \cdot 27 \pm 0 \cdot 028$ n = 10 (0·14–0·44) | This paper |
| | | | | Mouse 0·06–0·20 | Berk, Nelson and Pickett (1960) |
| | | | | Rabbit 0·4–0·91 | Harris and Barclay (1955) |
| | | Rabbit 0·078–0·16 | | | Cohn and Morse (1960) |
| | | Guinea-pig 0·23 | | Guinea-pig 0·62 | Stähelin, Suter and Karnovsky (1956) |

TABLE II.—*$QO_2$ µmoles $O_2$/mg. Dry Weight/hr.*

| | Mouse BP8 ascites tumour | Rat hepatoma ascites cells | Mouse polymorphonuclear leucocytes | Mouse lymphocytes | Mouse macrophages |
|---|---|---|---|---|---|
| Mean diameter (100 cells) | 16·15 | 16·78 | 11·14 | 5·60 | 10·42 |
| Calculated volume $\mu^3$ | 2200 | 2490 | 740 | 93 | 600 |
| Calculated net weight of $10^7$ cells in mg. | 23 | 26 | 7·8 | 0·98 | 6·3 |
| Estimated dry weight mg. | 4·6 | 5·23 | 1·56 | 0·2 | 1·26 |
| $QO_2$/mg. dry weight | 0·254 | 0·075 | 0·2 | 1·3 | 0·214 |

The $QO_2$ of BP8 ascites tumour cells by direct measurement of dry weight was 0·26. Aisenberg (1961) gave a $QO_2$ of 0·31 for Ehrlich ascites cells.

$QO_2$ was expressed as µmoles $O_2$/mg. dry wt/hr, as shown in Table II, the lymphocyte because of its small size gave the highest value.

At low $pO_2$'s the rate of oxygen uptake by the cells diminished, showing that oxygen concentration became a limiting factor. The ½ Vmax values (the apparent Michaelis constant for oxygen or $KmO_2$) were of the order of 1–5 mm. Hg, but as the system was rapidly altering these values must be regarded as qualitative only. (Longmuir used a polarographic method to find the $KmO_2$ of bacteria (1954) and of liver cells (1957), and Froese (1962) found a $KmO_2$ of 0·18 mm. Hg for Ehrlich ascites cells.) We found that low concentrations of lysolecithin per cell considerably increased the $KmO_2$ values with little decrease in Vmax. This type of effect is seen in competitive inhibition. It may indicate that these amounts of lysolecithin reduce oxygen uptake by damaging the mitochondria or by switching the metabolism from oxidative to glycolytic pathways. Coagulation was excluded as a cause of this effect.

At higher concentrations of lysolecithin per cell, the percentage inhibition of oxygen uptake increased and was found to be proportional to log [dose of lysolecithin per cell]. The values for 50% inhibition of all the cell types studied are listed in the first line of Table III. In terms of dose per cell rat hepatoma cells were the least sensitive, followed by polymorphonuclear leucocytes, BP8 cells, lymphocytes and macrophages. Fischer's (1964) claims could therefore not be confirmed, and his experiments have already been challenged as not truly representing the effect of lysolecithin itself on the cells.

TABLE III.—*Dose of Lysolecithin* $\mu g./10^6$ *Total Cells and in* $\mu g./\mu^2$ *of Cell Surface which Produced* 50% *Inhibition of Oxygen Uptake in Mouse and Rat Cells*

| | Mouse BP8 ascites tumour | Rat hepatoma ascites cells | Mouse polymorphonuclear leucocytes | Mouse lymphocytes | Mouse macrophages |
|---|---|---|---|---|---|
| 50% inhibition dose $\mu g.$ lysolecithin/$10^6$ total cells | $6 \cdot 4 \pm 0 \cdot 78$ | $11 \cdot 8 \pm 0 \cdot 81$ | $8 \cdot 4 \pm 0 \cdot 73$ | $4 \cdot 5 \pm 0 \cdot 31$ | $4 \cdot 24 \pm 0 \cdot 28$ |
| Mean area of 100 cells $\mu^2$ | $850 \cdot 7 \pm 29 \cdot 3$ | $906 \cdot 8 \pm 31 \cdot 1$ | $392 \cdot 9 \pm 7 \cdot 4$ | $102 \cdot 9 \pm 4 \cdot 3$ | $352 \cdot 9 \pm 12 \cdot 7$ |
| 50% inhibition dose per $\mu^2$ of cell surface $\mu g./\mu^2 \times 10^{-9}$ | $7 \cdot 5 \pm 0 \cdot 95$ | $13 \cdot 0 \pm 0 \cdot 99$ | $21 \cdot 4 \pm 1 \cdot 89$ | $43 \cdot 3 \pm 3 \cdot 53$ | $12 \cdot 0 \pm 0 \cdot 92$ |

Haemolysis of erythrocytes is complete with 0·3 $\mu g.$ (concensus of several references), area of human erythrocyte is 140 $\mu^2$ (Wintrobe, 1952) therefore lysolecithin $2 \cdot 15 \times 10^{-9}$ $\mu g./\mu^2$ gives complete haemolysis of erythrocyte.

Tumour cells, however, are considerably larger than the leucocytes studied; and since lysolecithin is a surface active agent, its activity must be expected to depend on the surface area of the cell involved. The diameters of 100 cells of each type were therefore measured under the phase contrast microscope in a hanging drop suspension. These data are shown in Table II, and the means and S.E.M. of the 100 areas calculated from the diameters are shown in Table III. The 50% inhibition dose in $\mu g.$ per square micron of cell surface was calculated, and on this basis the BP8 tumour cell was found to be most sensitive and the lymphocyte least sensitive to the action of lysolecithin.

This type of analysis we believe is justified by the results of those who have studied the effects of lysolecithin on red cells. For instance, Gorter and Hermans (1943) found that haemolysis occurred when enough lysolecithin was present on the surface of the cell to form a complete monolayer. Hughes (1935) gives 108 Å$^2$ for the molecular area of lysolecithin; and Klibansky and de Vries (1963) calculated from this figure that 0·1 $\mu g./10^6$ cells would be necessary for lysis, and showed that 0·01 $\mu g./10^6$ cells, measured by direct lipid analysis, caused sphering of erythrocytes. But they also found that only 20% of the lysolecithin in suspension became attached to the cell membrane, and, from the data of several other workers, a figure of 0·3 $\mu g./10^6$ cells emerges for haemolysis by the lysolecithin in suspension. (Gorter and Hermans (1943), Scarinci, Parenti, Cantone and Ravazzoni (1960); Hartree and Mann (1960), Phillips and Middleton (1965).) Taking the area of the erythrocyte as 140 $\mu^2$ (Wintrobe, 1952) gives a value of $2 \cdot 15 \times 10^{-9}$ $\mu g.$ lysolecithin per $\mu^2$ of cell surface for the haemolysis of erythrocytes. It will be seen that this value is much less than the $7 \cdot 5 \times 10^{-9}$ $\mu g./\mu^2$ required to produce 50% inhibition of oxygen uptake in BP8 tumour cells (Table III).

The effect of lysolecithin on erythrocytes is probably due to the formation of either lysolecithin-cholesterol complexes (Collier, 1952) or lysolecithin complexes with the lecithin of the cell membrane (Klibansky and de Vries, 1963). Resulting changes in the micellar shape of the discs of lecithin alter the surface activity of the red cell to produce sphering. The formation of such complexes would involve irreversible attachment of lysolecithin to the cell. The present experiments have confirmed this irreversibility for the cells studied, by showing that inhibition was completed rapidly and that once attached the lysolecithin was not available for other cells. However, the effect of lysolecithin on tumour cells and leucocytes is certainly more complex. Mouse lymphocytes, with rather less surface area than human erythrocytes, required lysolecithin in 15 times the haemolysis dose to produce 50% inhibition of the oxygen uptake. This dose caused distortion of the cell surface but complete lysis did not occur even with 20 $\mu$g./$10^6$ cells—66 times the dose required to produce haemolysis. It is possible that the red cell surface is more susceptible to structural damage which then proceeds to haemolysis, whereas a similar change in leucocytes may merely lead to increased permeability. Albumin is a powerful inhibitor of lysolecithin, and the white cells may be able to slough off proteins into the lysolecithin suspension.

Such changes in cell-surface properties would not necessarily bring about a decrease in oxygen uptake. However, intracellular actions of lysolecithin have also been reported. Particles which carry out oxidative phosphorylation are rich in phospholipids (Cooper and Lehninger, 1956), and *low* concentrations of lysolecithin protect phospholipids from salt precipitation (Saunders, 1957). Habermann (1954) found that lysolecithin inactivated the enzymic systems of oxidative phosphorylation in liver homogenates, and suggested that phospholipids played a role between respiration and phosphorylation. Witter, Morrison and Shepardson (1957) found that lysolecithin acted on the binding structures of the enzyme complex rather than on the individual enzymes: inhibition of oxidative phosphorylation would be expected if spatial disarrangement of the enzymes on the mitochondrial cristae occurred. They showed that lysolecithin uncoupled oxidative phosphorylation, decreased the oxidation of substrates and decreased the stability of mitochondria. They postulated that lysolecithin in low concentration played a part in the control of normal oxidative phosphorylation. Nygaard, Dianzani and Bahr (1954) observed disintegration of mitochondria and inactivation of succinoxidase systems. Seven minutes incubation was sufficient: 10 mg./ml. caused complete destruction of the suspension used, while 0·1 mg./ml. caused less than 10% destruction. Witter and Cottone (1956) observed swelling of isolated mitochondria with lysolecithin. Finally Hartree and Mann (1960) found that lysolecithin caused stimulation of oxygen consumption by ram spermatozoa at concentration less than 1 mM, but inhibition at concentrations greater than this.

In terms of the whole cell, therefore, it may be suggested that lysolecithin has two actions. The first is to increase the permeability of the membrane. This would allow entry of lysolecithin *into* the cell in sufficient concentrations to disorganize mitochondria and inhibit oxidative phosphorylation. If this were so, it would be suspected that there would be a minimum of lysolecithin needed per cell before any inhibition occurred. This was in fact observed; at concentrations less than 1 $\mu$g./$10^6$ cells, no inhibition occurred. (A similar minimum was observed for BP8 cells and lymphocytes: it was the *slope* of the inhibition curve that varied.) The stimulation of oxygen uptake observed in lymphocytes at low lysolecithin concentrations might be explained by entry of very small amounts into the cell, stabilizing the mitochondria according to the suggestion put forward by Saunders (1957).

With respect to a possibly greater action of lysolecithin on tumour cells than on normal cells, two observations are important. Ponder and Ponder (1964) found that extracts of tumour cells had a greater haemolytic activity than extracts of lung or liver. They postulated the existence of lysolecithin inhibitor, which was more strongly bound in normal tissues. Furthermore, Gray (1963) found that although there was no great increase of lysolecithin in Landschutz and BP8 ascites tumours, the membrane lipids had a less specific organization of fatty acids and a rather high proportion of breakdown products. He suggested that " the presence of substantial amounts of monoglyceride, phosphatidic acid and lysolecithin may reflect a tendency to general breakdown of tumour lipids, perhaps by enzymes released by changes in membrane permeability ". Both explanations would account for an increased sensitivity to lysolecithin.

In conclusion, care must be taken in extrapolating these results to conditions in solid tumours *in vivo*. Unknown factors—e.g. the amount of lysin-inhibitors such as albumin in the extracellular fluid, and the ability of lysolecithin micelles to diffuse through the extracellular space—must be taken into account. Furthermore, the effect of lysolecithin on tumour capillaries, and the possibilities of haemorrhage or increased vascular permeability (Cater and Taylor, 1966) may complicate the issue. The present results, however, have suggested that further work along such lines might be rewarding.

### SUMMARY

1. A method is described for measuring the oxygen uptake of cell suspensions *in vitro*, by means of Silver's oxygen cathode.
2. BP8 ascites and rat hepatoma cells had a higher respiratory rate on a per cell basis than polymorphonuclear leucocytes, lymphocytes, or macrophages.
3. Lysolecithin inhibited the oxygen uptake of all cell types studied, at concentrations greater than 1·0 $\mu$g./$10^6$ cells. The degree of inhibition was proportional to the logarithm of the dose of lysolicithin per cell. In terms of surface area, tumour cells were more sensitive than lymphocytes or polymorphonuclear leucocytes.
4. Concentrations of lysolecithin less than 1 $\mu$g./$10^6$ cells caused no inhibition of oxygen uptake. In two experiments, such concentrations stimulated the oxygen uptake of lymphocytes.
5. Low concentrations of lysolecithin caused an increase in the apparent $KmO_2$ value for the cells.
6. Lysolecithin-treated cells characteristically showed increased permeability to Trypan Blue, distortion of the cell membrane, and often coagulation.

We wish to thank Dr. I. A. Silver of the Department of Veterinary Anatomy, University of Cambridge, for the loan of two of his micro/membrane covered oxygen-cathodes.

### REFERENCES

AISENBERG, A. C.—(1961) 'The Glycolysis and Respiration of Tumours'. London (Academic Press).

BERK, R., NELSON, E. L. AND PICKETT, M. J.—(1960) *J. infect. Dis.*, **107**, 175.

CATER, D. B., SILVER, I. A. AND WILSON, G. M.—(1959) *Proc. R. Soc.* B. **151**, 256.

CATER, D. B. AND TAYLOR, C. R.—(1966) *Br. J. Cancer*, **20**, 517.

COHN, Z. A. AND MORSE, S. I.—(1960) *J. exp. Med.*, **111**, 667.
COLLIER, H. B.—(1952) *J. gen. Physiol.*, **35**, 617.
COOPER, C. AND LEHNINGER, A. L.—(1956) *J. biol. Chem.*, **219**, 489.
COTRAN, R. S. AND MAJNO, G.—(1964) *Ann. N.Y. Acad. Sci.*, **116**, 750.
DAWSON, R. M. C., MANN, T. AND WHITE, I. G.—(1957) *Biochem. J.*, **65**, 627.
FISCHER, H.—(1964) *Ann. N.Y. Acad. Sci.*, **116**, 1063.
FROESE, G.—(1962) *Biochim. biophys. Acta*, **57**, 509.
GORTER, E. AND HERMANS, J. J.—(1943) *Recl. Trav. chim. Pays-Bas. Belg.*, **62**, 681.
GRAY, G. M.—(1963) *Biochem. J.*, **86**, 350.
HABERMANN, E.—(1954) *Naturwissenschaften*, **41**, 429.
HANKS, J. H.—(1948) *J. cell comp. Physiol.*, **31**, 235.
HARRIS, H. AND BARCLAY, W. R.—(1955) *Br. J. exp. Path.*, **31**, 592.
HARTREE, E. F. AND MANN, T.—(1960) *Biochem. J.*, **75**, 251.
HUGHES, A.—(1935) *Biochem. J.*, **29**, 430.
KLIBANSKY, C. AND DE VRIES, A.—(1963) *Biochim. biophys. Acta.*, **70**, 176.
LESLIE, I., FULTON, W. C. AND SINCLAIR, R.—(1957) *Biochim. biophys. Acta.*, **24**, 365.
LONGMUIR, I. S.—(1954) *Biochem. J.*, **57**, 81.—(1957) *Biochem. J.*, **65**, 378.
MIDDLETON, E. AND PHILLIPS, G. B.—(1963) *Nature, Lond.*, **198**, 758.
— (1964) *J. Lab. clin. Med.*, **64**, 889.
NELSON, E. L. AND BECKER, J. R.—(1959) *J. infect. Dis.*, **104**, 13.
NYGAARD, A. P., DIANZANI, M. V., AND BAHR, G. F.—(1954) *Exp. Cell Res.*, **6**, 453.
PHILLIPS, G. B. AND MIDDLETON, E.—(1965) *J. Immunol.*, **94**, 40.
PONDER, E. AND PONDER, R. V.—(1964) *Nature, Lond.*, **204**, 995.
ROBINSON, N.—(1961) *J. Pharm. Pharmac.*, **13**, 321.
SAUNDERS, L.—(1957) *J. Pharm. Pharmac.*, **9**, 834.
SCARINCI, V., PARENTI, M. A., CANTONE, A. AND RAVAZZONI, C.—(1960) *Arch. int. Pharmacodyn.*, **123**, 472.
SILVER, I. A.—(1963) *Med. Electron. Biol. Engng*, **1**, 547.
STÄHELIN, H., SUTER, E. AND KARNOVSKY, M. L.—(1956) *J. exp. Med.*, **104**, 121.
WINTROBE, M. M.—(1952) ' Clinical Hematology '. 3rd edition. Philadelphia (Kimpton), p. 86.
WITTER, R. F. AND COTTONE, M. A.—(1956) *Biochim. biophys. Acta*, **22**, 372.
WITTER, R. F., MORRISON, A. AND SHEPARDSON, G. R.—(1957) *Biochim. biophys. Acta*, **26**, 120.